# Cytokinin-microbiome interactions regulate developmental functions

**DOI:** 10.1186/s40793-022-00397-2

**Published:** 2022-01-15

**Authors:** Rupali Gupta, Dorin Elkabetz, Meirav Leibman-Markus, Elie Jami, Maya Bar

**Affiliations:** 1 Department of Plant Pathology and Weed Research, Plant Protection Institute, Agricultural Research Organization, Volcani Institute, Rishon LeZion, Israel; 2 Department of Plant Pathology and Microbiology, Hebrew University of Jerusalem, Rehovot, Israel; 3 Department of Ruminant Science, Animal Science Institute, Agricultural Research Organization, Volcani Institute, Rishon LeZion, Israel

**Keywords:** *Bacillus*, Cytokinin, Microbiome, Morphogenesis, Plant growth promotion, Plant development

## Abstract

**Background:**

The interaction of plants with the complex microbial networks that inhabit them is important for plant health. While the reliance of plants on their microbial inhabitants for defense against invading pathogens is well documented, the acquisition of data concerning the relationships between plant developmental stage or aging, and microbiome assembly, is still underway. The plant hormone cytokinin (CK) regulates various plant growth and developmental processes. Here, examining the relationships between plant development and microbiome assembly, we observed developmental-age dependent changes in the phyllopshere microbiome. We show that age-related shifts in microbiome content vary based on content of, or sensitivity to, CK.

**Results:**

We found a developmental age associated decline in microbial richness and diversity, accompanied by a decline in the presence of growth promoting and resistance inducing Bacilli in the phyllosphere. This decline was absent from CK-rich or CK-hypersensitive genotypes. *Bacillus* isolates we obtained from CK rich genotypes were found to alter the expression of developmental genes to support morphogenesis and alter the leaf developmental program when applied to seedlings, and enhance yield and agricultural productivity when applied to mature plants.

**Conclusions:**

Our results support the notion that CK supports developmental functions in part via the bacterial community.

**Supplementary Information:**

The online version contains supplementary material available at 10.1186/s40793-022-00397-2.

## Background

The phyllosphere microbial community plays positive roles in host plant life. Disease resistance, abiotic stress tolerance, improved vigor and alterations in life cycle phenology have been documented in the presence of specific bacterial communities [1, 2]. The plant leaf niche occupies a large surface area, and is important for the structure and function of the plant microbial community [3]. The agricultural and ecological implications of plant-beneficial interactions with microbes have motivated intense investigation into the factors that shape phyllopshere microbiota [4]. Deciphering the factors underlying the composition and dynamics of microbiome assembly is a key step towards understanding how the microbial community affects plant health and development.

In terms of diversity and richness, the phyllosphere hosts complex microbial communities that are determined by several dynamic factors, such as plant age, plant genotype, environmental variables, geographical location and agricultural practices [5, 6]. Previous work has uncovered factors that are central in determining the composition of microbiota. In particular, plant genotype has been identified to be an important driver that influences the structure of the phyllopshere microbiome [7, 8]. In addition to host genotype, geographic growth location has also been defined as a dominant factor influencing community structure. For instance, perennial plants belonging to the same species grown in different geographic locations showed surprisingly similar leaf microbial communities than different plant species grown in close proximity [9]. Recently, Li et al. [10] found that the growth stage and genotype of *Arabidopsis thaliana* are crucial in shaping phyllosphere bacterial composition, with the former being a stronger driver. Many studies on the structure of plant-associated microbial communities have shown that plants grown in sterile conditions house microbes that resemble airborne communities, while plants grown in natural conditions often have phyllosphere communities comprised of soil microbiota [7, 11]. Thus, from previous studies it becomes evident that the phyllosphere microbiome structure is complex, being influenced by various dynamic factors.

Growth stage- or age dependent bacterial community shifts in the rhizosphere have been well documented [12, 13]. The phyllosphere microbiome also undergoes dynamic changes as plants develop and/or age, as shown in Arabidopsis [14], *Lactuca sativa* [15] and *Boechera stricta* [8]. These age-related shifts in microbial content are presumably linked with age-dependent changes in the plant, such as hormonal and / or physiological variation. Plant aging in *B. stricta* differentially affected the abundance of multiple leaf-associated microbial taxa when grown at different geographical sites [8]. In the phyllopshere, age-related microbiome differentiation may be associated with the differences in the leaf structure or geometry, cuticle structure, trichome placement, or composition of the volatile substances secreted by the leaf. We recently reported that leaf structural niches influence phyllosphere microbial content in different genotypes [16].

Plant age and developmental status are important factors influencing host immune responses [9, 14, 17]. Plants have been shown to have differential age-dependent immune responses at the organ level [18]. In *A. thaliana*, young rosette leaves exhibit greater salicylic acid (SA) accumulation and SA-mediated resistance than older rosette leaves [18]. Age-dependent fluctuations in host resistance can assist plants in prioritizing the protection of valuable tissues, such as young leaves [19]. However, little is known about the relationships between plant growth regulation hormones, plant developmental stage, and bacterial communities in the phyllosphere.

The plant hormone cytokinin (CK) regulates various developmental processes, including embryogenesis, cell division and differentiation, shoot and root apical meristem maintenance, shoot and root lateral organ formation, and many others [20–22]. Thus, it is not surprising that changing endogenous CK content or signaling would cause alterations to plant development, resulting in changes to organ structure and patterning. CK has been demonstrated to promote morphogenesis and delay differentiation during plant development in many different plant species and developmental contexts [23–27] likely by delaying the differentiation of meristematic cells [28]. Tomato plants with altered CK content have altered developmental programs, and modified organ structures. Overexpressing the CK biosynthesis gene *ISOPENTENYL TRANSFERASE7* (*IPT7*), resulting in elevated endogenous levels of CK [29], or mutating in the MYB transcription factor *CLAU*, resulting in increased CK sensitivity [30], both result in highly patterned and complex leaves. Concomitantly, decreasing endogenous levels of CK by overexpressing *CK OXIDASE*3 (*CKX3*) [29], results in simplified leaves bearing less organs [31].

Recently, we investigated the relationship between CK and the phyllosphere microbiome in tomato, demonstrating that CK acts as a selective force in microbiome assembly, increasing richness, and promoting the presence of Firmicutes [16]. We found CK-mediated immunity to partially depend on the microbial community. In our previous work, we characterized several bacilli isolates from different species obtained from the phyllosphere of tomato having a high cytokinin content (*pBLS* >> *IPT)*, and found that these bacilli promote plant immunity and disease resistance [16]. Using biomimetics, we found that tomato genotypes with high CK content or increased CK sensitivity are better able to support bacilli in their phyllosphere, due to the altered leaf structures present in theses genotypes.

Our previous study defined CK as a driving force in microbiome assembly, and demonstrated that CK-mediated immunity is dependent in part on the microbiome. The idea that hormonal cues can be mediated by the microbiome warranted further investigation. Thus, we hypothesized, that CK-mediated developmental processes might also be dependent on the microbiome. To examine this, in the present study, we investigated developmental-age dependent changes in the microbiome. We explored whether age-related shifts in the microbiome are affected by CK content / sensitivity. Our results suggest that CK-dependent developmental functions are mediated via the bacterial community.

## Results

### Effects of plant developmental status on the phyllosphere microbiome

**Fig. 1:**
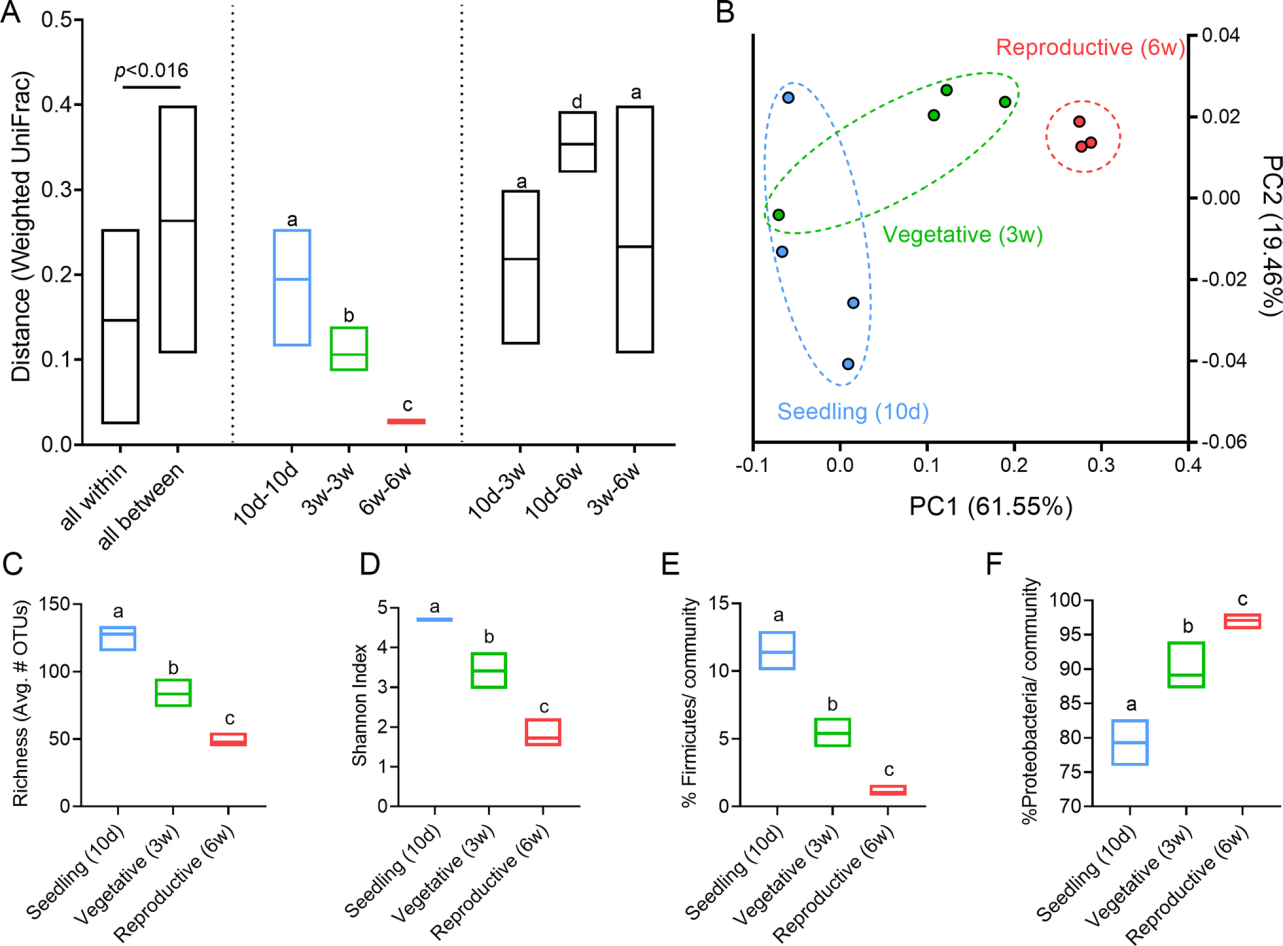
Developmental aging is accompanied by a decrease in bacterial community diversity, richness, and Firmicute content. 16S rRNA sequencing of the bacterial phyllosphere of randomly interspersed *S. lycopersicum* M82 plants grown in a net house in the winter of 2018, N = 4 for each genotype, of different ages: "Seedling" (10 days old), "Vegetative" (3 weeks old), and "Reproductive" (6 weeks old). **A** Weighted UniFrac beta diversity. Distance is significantly smaller within groups then between groups (*p* < 0.016). **B** Principal coordinates analysis of distance between all individual samples in the weighted UniFrac beta diversity calculations. **C** Species richness- alpha diversity. **D** Shannon index. **E** Proportion of Firmicutes in the bacterial community of indicated genotypes. **F** Proportion of Proteobacteria in the bacterial community of indicated genotypes. Floating bars encompass minimum to maximum values, line indicates mean. Different letters indicate statistical significance between samples in a two-tailed *t*-test with Welch's correction. **C** *p* < 0.0073; **D** *p* < 0.04; **E** *p* < 0.0045; **F** *p* < 0.0007

To examine the effect of plant developmental stage on phyllosphere composition, microbial DNA was prepared from the phyllosphere of randomly interspersed tomato (*Solanum lycopersicum* M82) plants of various developmental ages. When examining community structure between the samples using weighted UniFrac distances, we observed a significant clustering of the samples based on their developmental stage, demonstrating that the distance among biological replicates were significantly smaller within groups then between groups (Fig. 1A, B). Interestingly, distances between the samples of the same age also decreased in parallel to the increase in developmental age, with the smallest distance observed in the oldest, reproductive group. Community richness (Fig. 1C), Shannon index (Fig. 1D), and proportion of Firmicutes in the bacterial community (Fig. 1E), also all decreased as developmental age increased, while the proportion of Proteobacteria in the bacterial community, primarily Gammaproteobacteria, increased with aging (Fig. 1F).

**Fig. 2:**
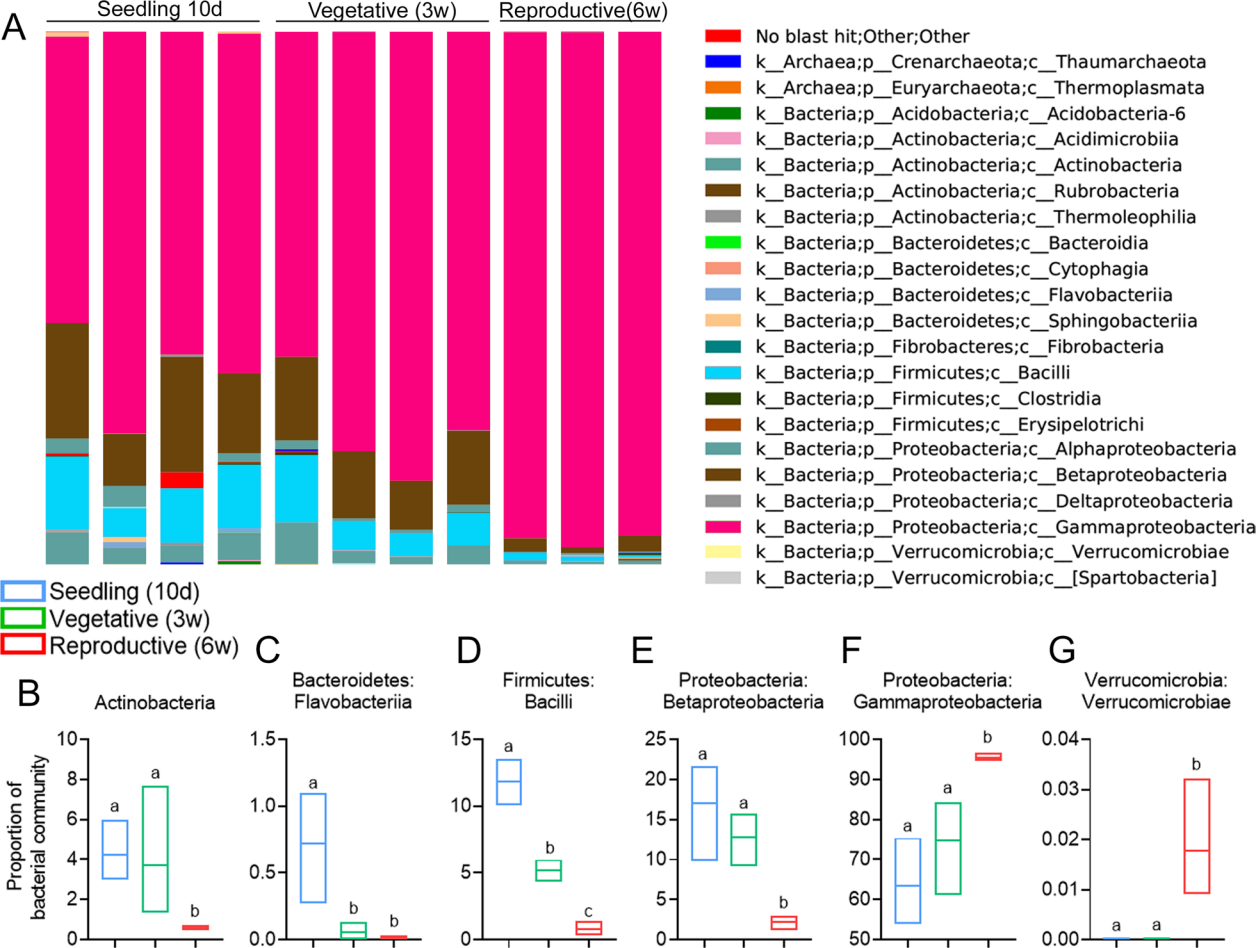
Changes to key bacterial OTUs in the microbial community over the course of aging. Representation of different key bacterial OTUs found in 16S rRNA sequencing of different plant ages: "Seedling" (10 days old), "Vegetative" (3 weeks old), and "Reproductive" (6 weeks old). **A** Stacked bars representing the percentage of key bacterial OTUs in the microbial community of individual plants of different ages as indicated. **B**–**G** Proportion of different key groups, as indicated, with statistical analysis. Floating bars encompass minimum to maximum values, line indicates mean. Different letters indicate statistically significant differences between samples in a two-tailed *t*-test with Welch's correction (**B**–**F**), or a Mann–Whitney test (**G**). **B** *p* < 0.0079; **C** *p* < 0.044; **D** *p* < 0.004; **E** *p* < 0.0076; **F** *p* < 0.018; **G** *p* < 0.029

Analyzing key bacterial OTUs in the microbial community over the course of aging, demonstrates age-relates shifts in many groups (Fig. 2), with gamma-proteobacteria increasing during the course of developmental aging, starting at about 65% relative abundance of the community in seedlings, and reaching over 95% relative abundance of the community in reproductive stage plants (Fig. 2A, F). This appears to occur "at the expense" of many other groups, the relative abundance of which decreased throughout aging, viz., Actinobacteria (Fig. 2B), Flavobacteria (Fig. 2C), bacilli (Figs. 1E, 2D), and Beta-Proteobacteria (Fig. 2E). Other groups, e.g., Verrucomicrobia (Fig. 2G), although present in very small amounts, are acquired only at the reproductive stage.

### The amount of bacilli in the bacterial community changes throughout development in a CK dependent manner

**Fig. 3:**
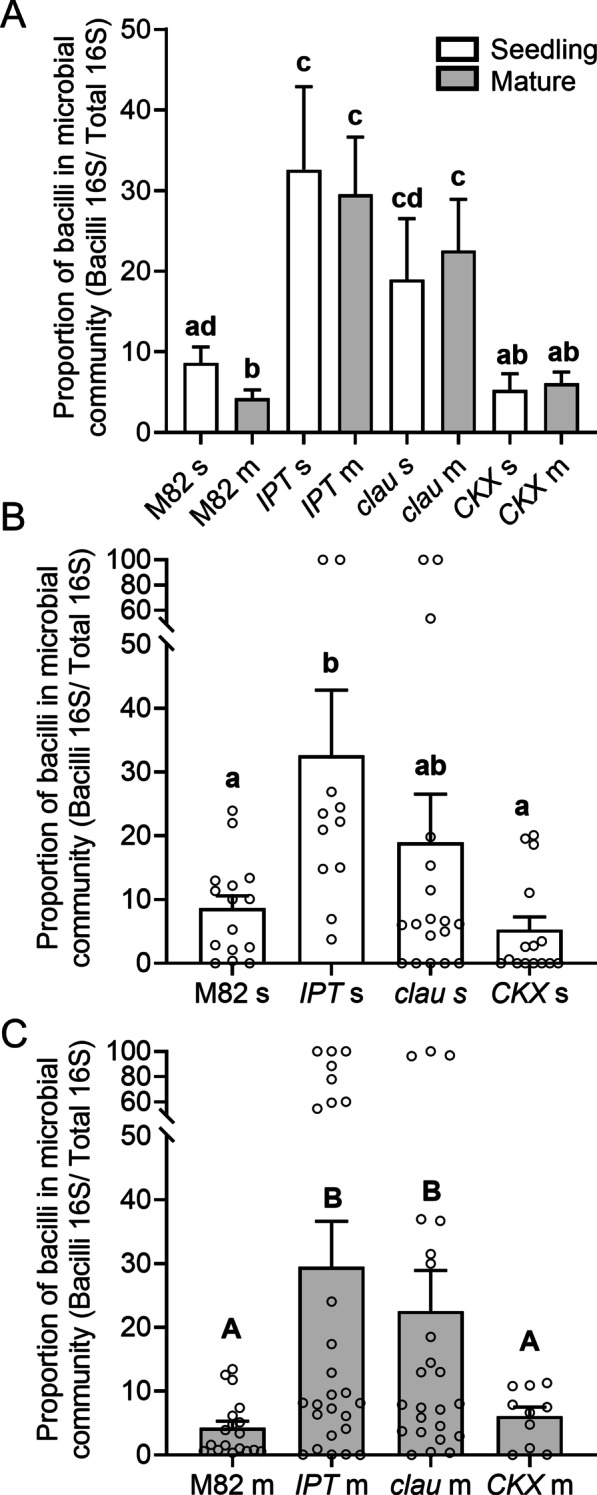
CK prevents age-associated decrease in bacilli content in the bacterial community. Bacterial DNA was extracted from indicated genotypes at the seedling (15-day old, indicated with "s" and white bars) and mature plant (45-day old, indicated with "m" and gray bars) stages. Bacilli amounts were estimated by qPCR of the bacillus 16S rRNA gene, normalized to qPCR of the total 16S rRNA gene content, and expressed as % of the total bacterial community. **A** Comparison of bacilli content in the background M82, the high CK content IPT, the high CK sensitivity *clau*, and the low CK content CKX, at both seedling and mature stages. **B** Comparison of the different genotypes at the seedling stage, all points shown. **C** Comparison of the different genotypes at the mature stage, all points shown. Bars depict mean ± SE. Different letters indicate statistically significant differences in an unpaired two-tailed *t*-test with Welch's correction, N > 10. **A** *p* < 0.048, **B** *p* < 0.043, **C** *p* < 0.018

We previously demonstrated that high CK content, or increased CK sensitivity, both support an increase in phyllosphere community richness, Shannon index, and in the proportion of Firmicutes [16]. Generally, CKs are thought to be synthesized mainly in the roots and transported via the xylem to the shoots, where they exert developmental functions [32, 33]. We hypothesized that the increased numbers of bacilli present in the bacterial community in seedlings (Fig. 1) may be supported by the increased levels of CKs present in young leaves, levels which decline over time [34], in accordance with the age-related decrease in Firmicutes we observed (Fig. 1E). We examined this by assaying the amount of bacilli present in the bacterial community in seedlings and mature plants, in M82 and high and low CK content genotypes, overexpressing *pBLS* >> *IPT* or *pFIL* >> *CKX,* as well as in the high CK sensitivity mutant *clausa*. As shown in Fig. 3, while bacilli percentages in the bacterial community decrease with developmental aging in M82, in the altered CK genotypes, the percentage in the microbial community does not change with age. *pFIL* >> *IPT* and *clausa* have increased percentages of bacilli in the microbial community in both the seedling (Fig. 3A, B) and mature [16], (Fig. 3A, C) stages, and, unlike in the background M82 (Figs. 1, 3A), the proportion of bacilli in the bacterial community does not decrease in mature plants when compared with seedlings [16]. We also observed higher amounts of microbial DNA in *pBLS* >> *IPT*, and lower amounts in *pFIL* >> *CKX*, suggesting that these genotypes also support increased or decreased amount of bacteria in general, respectively (Additional file 1: Fig. S1).

### Phylloshpere isolated bacilli from high-CK genotypes accelerate development

**Fig. 4:**
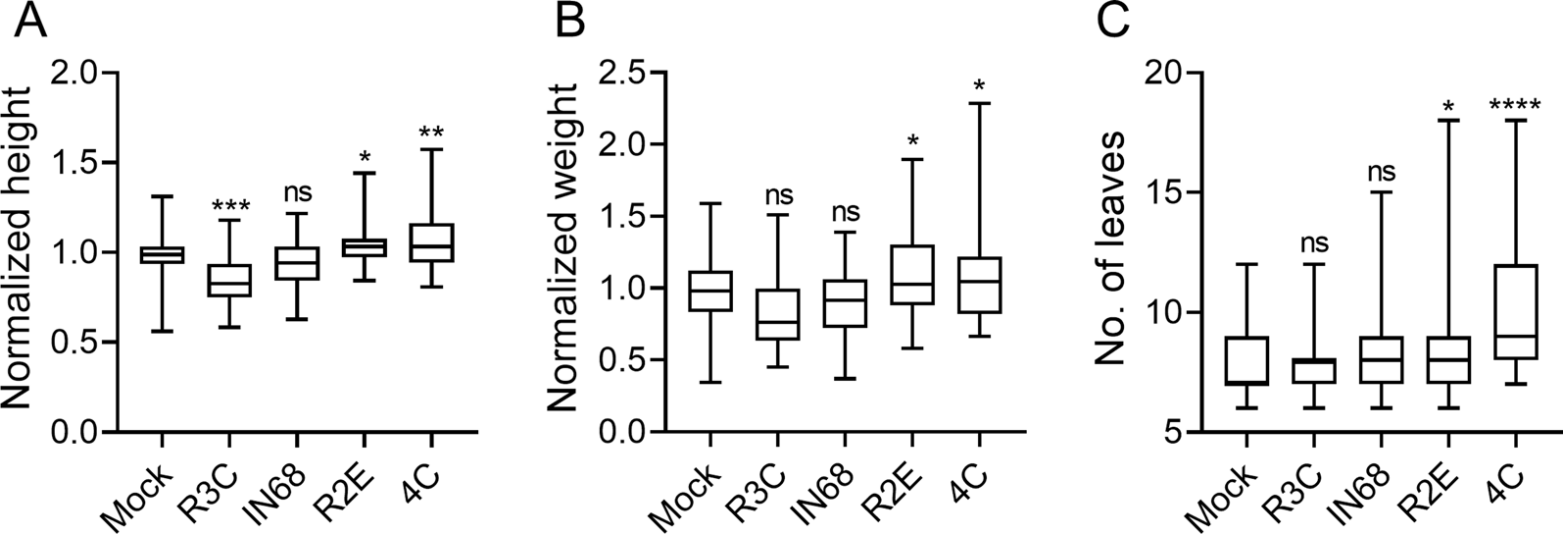
Bacilli isolated from high-CK genotypes affect development in seedlings. *S. lycopersicum* cv. M82 seedlings were treated with indicated bacteria (OD_600_ = 0.1), once a week for 2 weeks, starting from cotyledon emergence. Developmental parameters were measured in 10 day old M82 mock and bacterial isolate treated seedlings. **A** Seedling height (root crown to main shoot apical meristem) in centimeters. **B** Seedling weight. **C** Number of leaves produced starting from P1 (all initiated leaves were counted by dissecting the shoots). Boxplots depict minimum to maximum values, with box indicating inner quartile ranges and whiskers representing outer quartile ranges. Lines in box indicates median. Five independent experiments were conducted. Asterisks represent statistical significance from mock treatment in a one-way ANOVA with a Tukey post-hoc test (**A**, **B**), or a two-tailed *t*-test with Welch's correction (**C**). *p < 0.05; **p < 0.01; ***p < 0.001; ****p < 0.0001, ns = non significant. **A** N = 65, *p* < 0.041. **B** N = 50, *p* < 0.047. **C** N = 70, *p* < 0.029

Bacilli are well known to have growth-promoting effects [35]. Seedlings rapidly generate new organs, grow at a faster pace than mature plants, and have higher CK content than mature plants. Above, we observed that seedlings also support more bacilli than mature plants. Therefore, we next investigated whether our phyllosphere bacilli isolates could affect seedling development. We examined the early development of tomato seedlings following treatment with different bacterial isolates. We found that two bacterial treatments, one at cotyledon emergence, and the second after one week, were sufficient to induce accelerated growth and generation of leaves in the treated seedlings, in the case of the two bacilli isolates, *B. pumilus* R2E and *B. megaterium* 4C (Fig. 4A–C). This treatment regimen also had a negative effect on plant height in the case of the *Ralstonia* isolate R3C (Fig. 4A). Differentiation of the shoot apical meristem (SAM) to floral meristem and sympodial meristem was also significantly increased with 4C treatment (Additional file 1: Fig. S2).

**Fig. 5:**
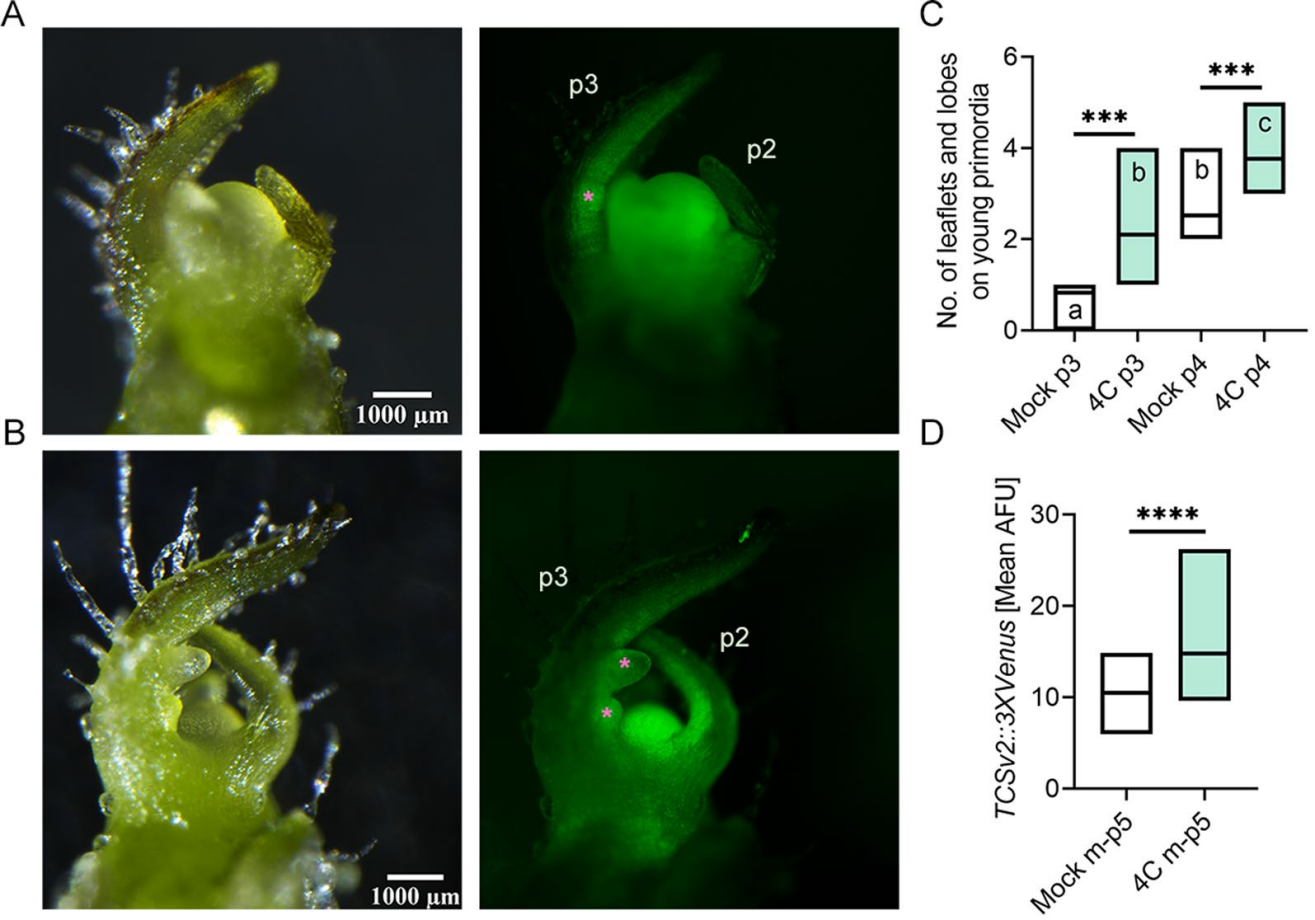
*B. megaterium* 4C accelerates leaf development and increases CK pathway activation. *S. lycopersicum* cv. M82 seedlings were treated with indicated bacteria (OD_600_ = 0.1), once a week for 2 weeks, starting from cotyledon emergence. Leaf complexity and TCSv2::3XVenus expression were measured in 10 day old M82 mock and 4C treated plants. **A** Typical Mock treated shoot apical meristem (SAM) and three youngest leaf primordia (p1–p3). Bar = 1000 µm. **B** Typical *B. megaterium* 4C treated SAM and p1-p3. P2 and p3 are indicated in the Venus fluorescence images, with asterisks indicating the nascent leaflets on p3. **C** Number of leaflets and lobes produced on p3 and p4. **D** TCSv2 driven total Venus fluorescence was measured as mean arbitrary fluorescent units (AFU) in images captured under identical conditions in shoots comprising the 5 youngest primordia. Each primordia was quantified for leaflet number and TCS expression when Leaf No. 5 was at the indicated developmental stage- all quantifications were done on the fifth leaf as it developed. **C**, **D** Floating bars depict minimum to maximum values, with lines indicating mean. Three independent experiments were conducted. Asterisks represent statistical significance from mock treatment, and different letters represent statistically significant differences among samples, in a one-way ANOVA with a Dunnett post-hoc test (**C**), or in a two-tailed *t*-test (**D**). ***p < 0.001; ****p < 0.0001. **C** N = 12, *p* < 0.0002. **D** N = 21, *p* < 0.0001

In addition to the growth and organ initiation promoting effects we observed (Fig. 4), changes to the plant developmental program following bacterial treatment were an intriguing possibility. Treatment with bacterial isolates increased the number of leaves produced (Fig. 4C). We therefore next conducted an in depth analysis of leaf development, which follows a predictable and well characterized program *in S. lycopersicum* M82 [26, 29], following treatment with *B. megaterium* isolate 4C. We found that starting from p3, *B. megaterium* 4C treatment results in a significant increase in leaf patterning (Fig. 5A–C). Leaf complexity over time was also examined upon *B. megaterium* 4C or *B. pumilus* R2E treatment in seedlings (Additional file 1: Fig. S3). We found that both R2E and 4C increase leaf complexity (Additional file 1: Fig. S3), with 4C doing so earlier. Leaf complexity significantly increased during development in the mock treated plants when comparing the first and last time points among mock treatments, as expected, reflecting the "normal" developmental program.

### Bacillus treatment activates the CK response machinery and developmental genes

Using the CK activity response synthetic promoter TCS (two-component signaling sensor) fused to the VENUS fluorescent protein as a reporter [36], we determined that the CK pathway was activated following bacterial treatment. When examining expression of the synthetic CK-responsive promoter *TCSv2* driving Venus in transgenic M82 tomato plants stably expressing *pTCSv2::3* × *VENUS*, we observed a significant increase in CK responsiveness of the leaf tissue in seedlings treated with *B. megaterium* 4C (Fig. 5A, B, D), indicating that the accelerated development correlates with an increase in CK pathway activation.

**Fig. 6:**
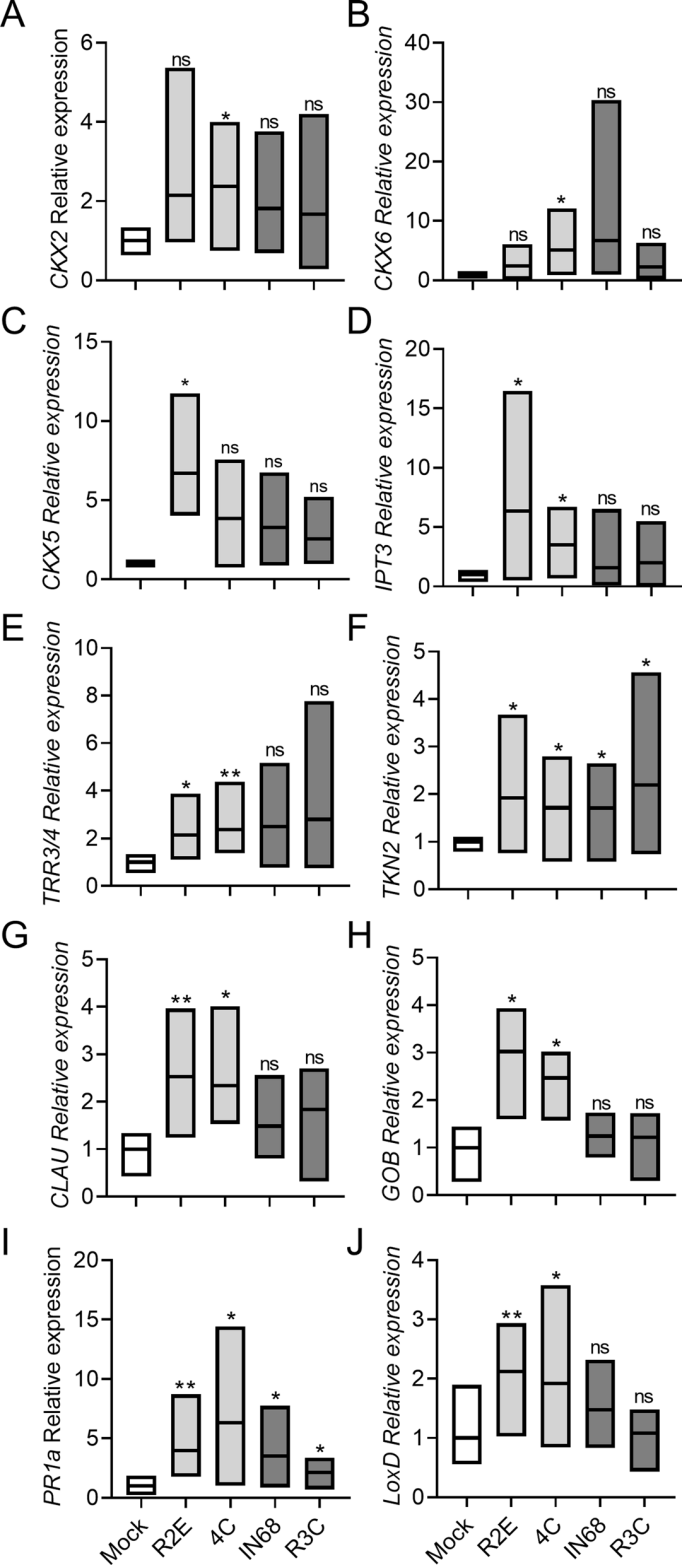
Bacilli from high-CK genotypes differentially activate morphogenetic genes and CK pathway genes. *S. lycopersicum* cv. M82 plants were pre-treated with indicated bacteria (OD_600_ = 0.1) once a week, two treatments in total, starting from cotyledon emergence. Gene expression was assayed by qRT-PCR, 3 days after the second treatment. Floating bars indicate minimum to maximum values (box) with mean (line in box). Bacilli are indicated in pale gray bars, gram negative bacteria in dark gray bars. Genes were normalized to a geometric mean of the expression of 3 normalizers: *SlExp*, *SlCYP*, and *SlRPL8*. **A** *SlCKX2*; **B** *SlCKX6*; **C** *SlCKX5*; **D** *SlIPT3*; **E** *SlTRR3/4*; **F** *SlTKN2*; **G** *SlCLAU*; **H** *SlGOB;* **I** *SlPR1a*; **J** *SlLoxD*. Asterisks indicate statistical significance from Mock treatment in an unpaired two-tailed *t*-test with Welch's correction, N = 6, *p* < 0.05. (*p value < 0.05; **p value < 0.01; ns- non significant)

To further characterize the effect of bacterial isolates on development, we next examined the expression of a variety of developmental genes. We found that the bacilli isolates exclusively activated CK pathway genes (Fig. 6A–E), with *B. megaterium* 4C not surprisingly activating more CK pathway genes than *B. pumilus* R2E. The expression of CK-responsive type-A tomato response regulators (*TRR*s) increased and the expression of *CKX* genes was also significantly altered (Fig. 6A–C). All isolates activated the meristem maintenance KNOTTED1-LIKE HOMEOBOX (KNOXI/*TKN2*) (Fig. 6F) [37, 38], while only the bacilli activated the differentiation MYB transcription factor *CLAU* (Fig. 6G) [30], and the organ boundary determination CUC transcription factor *GOB* (Fig. 6H) [39]. To verify the response to bacterial treatment, we examined SA pathway activation using *PR1a* (Fig. 6I) [22], and jasmonic acid (JA) pathway activation using *LoxD* (Fig. 6J) [40]. We found that all isolates activated the SA pathway (Fg. 6I), however, interestingly, only the bacilli isolates activated the JA pathway (Fig. 6J).

### Phylloshpere isolated bacilli from high-CK genotypes promote growth and increase agricultural productivity in mature plants

**Fig. 7:**
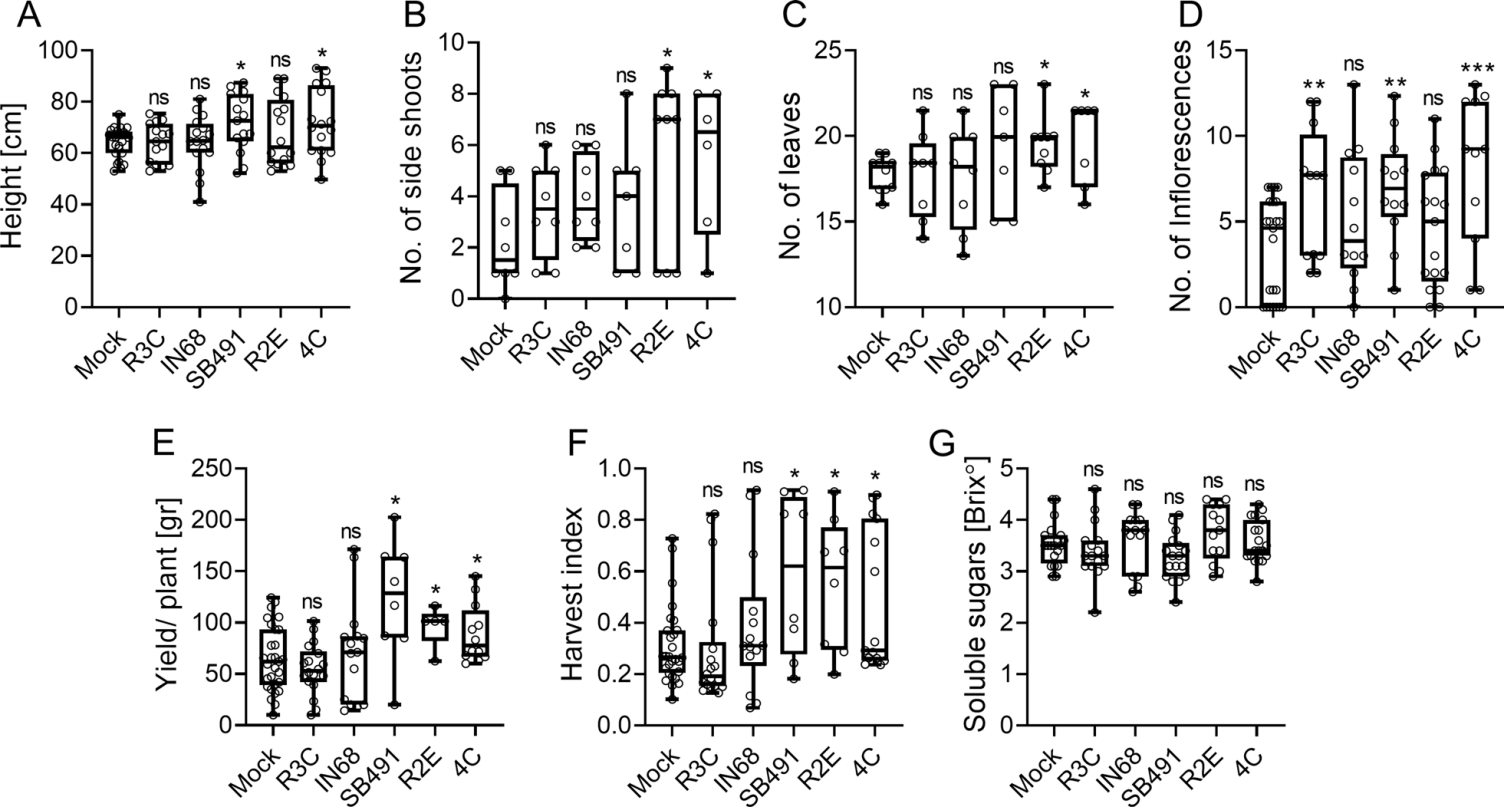
Bacilli from high-CK genotypes increase agricultural productivity. 3 week-old *S. lycopersicum* cv. M82 plants were treated with indicated bacteria (OD_600_ = 0.1), once a week for 4 weeks. Agricultural parameters were measured in M82 mock and bacterial isolate treated plants, just prior to harvest (65–75 days after germination). **A** Plant height (root crown to main shoot apical meristem) in centimeters. **B** Number of side shoots used as a measure of apical dominance. **C** Number of leaves produced. **D** The average number of inflorescences per plant. **E** Average yield, expressed as the total fruit weight per plant in grams. **F** Harvest index (HI), calculated as the ratio between the total mass of fruit yield and the total biomass. **G** Total soluble sugars were measured using a refractometer and are expressed as °Brix. Boxplots depict minimum to maximum values, with box indicating inner quartile ranges and whiskers representing outer quartile ranges. Lines in box indicates median. Four independent experiments were conducted. Asterisks represent statistical significance from mock treatment in a two-tailed *t*-test with Welch's correction, *p < 0.05; **p < 0.01; ***p < 0.001. **A** N = 16, *p* < 0.035. **B** N = 8, *p* < 0.033. **C** N = 8, *p* < 0.05. **D** N = 12, *p* < 0.0095. **E** N = 9, *p* < 0.037. **F** N = 9, *p* < 0.043. **G** N = 12, ns = non significant

Since we found that bacilli isolates from high-CK genotypes accelerated development and growth in seedlings, we next examined whether they could affect growth and agricultural productivity in older plants. Several instances of agricultural use for bacilli have been reported [35]. Treatment with the* Bacillus* isolate *B. megaterium* 4C increased plant height (Fig. 7A) and decreased apical dominance (Fig. 7B), increased the number of leaves (Fig. 7C), number of inflorescences (Fig. 7D), as well as the average yield per plant (Fig. 7E) and harvest index (Fig. 7F). Treatment with the bacillus isolate *B. pumilus* R2E decreased apical dominance (Fig. 6B), and increased the number of leaves (Fig. 7C), as well as the average yield per plant (Fig. 7E) and harvest index (Fig. 7F), but did not affect plant height (Fig. 7A) or the number of inflorescences (Fig. 7D). The gram negative controls, *R. picketti* R3C and *Pseudomonas aeruginosa* IN68, had no effect on agricultural parameters, except for an increase in the number of observed inflorescences with IN68 (Fig. 7D). A control *B. subtilis* lab strain, SB491, also increased some of the tested agricultural parameters vis., height (Fig. 7A), number of inflorescences (Fig. 7D), yield (Fig. 7E), and harvest index (Fig. 7F). None of the bacterial strains significantly affected fruit sugar content (Fig. 7G).

## Discussion

### Developmental stage influences the phyllopshere microbial community

Studies of the driving forces underlying microbial community formation have revealed that both environmental variables [6], and host genotype and age, can be defined as the key factors driving community content and assembly [7, 10], depending on the context of the study. Despite recent progress in our understanding of the phyllosphere microbiome, much of the variation found in the phyllosphere remains unsolved, suggesting that the driving forces shaping microbial community structure and function have not yet been adequately defined.

Our study considered the effect of plant developmental stages on microbial community composition of the phyllosphere microbiome. Analysis of the bacterial phyllosphere community dynamics throughout plant developmental stages revealed significant changes to community richness and diversity (Fig. 1). These results are in agreement with earlier reports concerning succession of microbial communities in the phyllosphere [8, 41, 42]. A more detailed look at the assembled phyllopshere microbiome through developmental aging revealed that the core microbiome, composed mostly of Firmicutes and Proteobacteria, was altered in the microbial communities across development, suggesting that changes in the conditions required for survival, succession, persistence, and colonization of different microbial taxa, may occur during plant development and growth. These factors were previously reported to be important in determining phyllosphere microbial content [11].

### Age-related changes to the microbiome are affected by CK content

CK is a central driver of development and morphogenesis. CK positively regulates cell division and proliferation in the plant shoot, driving mitosis and cytokinesis, and is involved in the organization of the shoot apical meristem [20, 43]. CK promotes morphogenesis by delaying the differentiation of meristematic cells [28]. Alterations to the CK pathway in tomato result in changes in leaf phenotypes [31]. Overexpression of the CK biosynthesis gene *AtIPT7* in tomato leaves leads to the formation of highly complex leaves, whereas overexpression of the CK oxidase/degradation gene *CKX*, results in reduced leaf complexity [29, 30].The *CLAU* gene promotes an exit from morphogenesis by negatively affecting CK signaling, resulting in increased leaf complexity upon its knockout in the *clausa* mutant [30]. Recently, we found that increased CK content, as in *pBLS* >> *IPT7,* or sensitivity, as in *clausa,* can have a strong effect on shaping the microbiome [16]. We found that high CK content or signaling increased species richness while reducing distances among samples within high-CK genotypes, resulting in dominant and consistent driving forces on bacterial community structure, and favoring Gram-positive bacteria, and bacilli in particular [16]. CK was found to shape the microbiome through both structural cues, with CK-patterned leaf structures resulting in niches that are favored by bacilli, and chemical cues, with CK promoting growth of some bacilli in vitro [16].

Here, we analyzed the content of phyllosphere microbiota from genotypes with different CK content or sensitivity, at the seedling and mature reproductive developmental ages. We found that the abundance of total bacilli was lower in wild type (WT) *M82 and pFIL* >> *CKX* than in *pBLS* >> *IPT* and *clausa* at both developmental stages (Fig. 3). The mature plant results were similar in our previous microbiome analysis of these genotypes [16]. Interestingly, examination of the microbiome shift between these two developmental stages with respect to bacilli content in high-CK and low-CK genotypes showed that, while bacilli spp. content decreases with age in the background M82, bacilli remain in high amounts, and do not decline with age, in the CK-rich genotypes *pFIL* >> *IPT* and *clausa* (Fig. 3A). We previously reported that CK can support bacilli [16] in the phyllosphere. Since mature plant tissues contain significantly lower levels of CK than seedlings [32, 33], the possibility arises that the age-related decline in bacilli in the microbiome of WT plants is due to the age-related decline in CK content [32].The increased levels of CKs present in seedlings might support the increased numbers of bacilli present in the microbial community in seedlings. These increased CK levels decline over time as the plant matures [44], which could explain the parallel decline in bacilli in the microbial community that we observed in the mature plants. This points to the possible existence of a specific microenvironment associated with CK levels, which distinctly promotes an abundance in bacilli. This microenvironment could be both structural, i.e., smaller cells and increased number of trichomes in young leaves when compared to mature leaves [45, 46], that create an altered physical topography available for bacterial colonization, as we previously reported in connection with high-CK genotypes [16], or direct chemical effects stemming from the actual changes in CK levels. Of note, is that we previously found CK was able to support our phyllopshere isolates in vitro, improving their growth, biofilm formation, and swarming motility, when applied in the absence of a plant [16]. Therefore, although distinct changes in structural leaf microenvironments available for colonization between seedling leaves and mature plant leaves are highly likely, it is also possible that the abundance of CK present in younger plants acts chemically to support an increased amount of bacilli in the phyllosphere.

### Bacilli isolated from high-CK genotypes can alter developmental programs and increase plant productivity

The microbiome has been demonstrated to be required for achieving predictable developmental outcomes, as plants in sterile or axenic environments often grow more slowly and have altered development [47, 48]. Recently, the necessity of plant growth promoting bacilli in the microbial community for disease management has been restated [49]. *Bacillus* spp. are well known to have plant growth promoting activities [35]. We reported that bacilli we isolated from *pBLS* >> *IPT* enhance disease resistance by triggering plant immunity [16]. A trade-off between induced disease resistance and plant growth has also been reported [14, 50]. The bacilli isolates we obtained from high-CK plant genotypes protected tomato plants from disease [16], while also supporting growth (Fig. 7), suggesting a positive, rather than negative, correlation between growth and defense when tomato plants are treated with these bacteria. This could indicate an agricultural advantage to treatment with certain bacilli isolates in specific regimens, and will be investigated further.

Our results indicate that the growth promotion exerted by bacilli strains isolated from high-CK environments are the result of alterations to developmental programs (Figs. 4and5, Additional file 1: S2-S3). During leaf development, the young leaf undergoes morphogenesis and reaches the mature, differentiated stage of its development simultaneously with the decline in its morphogenetic potential. Compound leaves of tomato are composed of multiple leaflets, which initiate basipetally from a meristematic region at the leaf margin known as the marginal blastozone [29, 51, 52]. The leaf morphogenetic potential is harbored by meristematic cells, which respond to CK and therefore exhibit *TCS* activation. *TCSv2* driven expression was observed in an expanded region in *B. megaterium* 4C treated tomato seedlings in the shoot apical meristem (SAM) and three youngest leaf primordia (p1-p3), demonstrating that CK pathway activation during leaf development was increased upon *B. megaterium* treatment (Fig. 5A, B, D). In parallel, leaves from bacillus treated plants displayed an increase in patterning, exhibiting 1–2 additional organs than typically observed on leaves of a similar developmental plastochron (Fig. 5A–C), confirming that morphogenesis is indeed promoted by *B. megaterium* 4C treatment.

CK pathway genes regulate the activity of meristems [28]. The KNOXI gene *Tkn2* plays important role in promoting leaf morphogenesis by delaying differentiation, preserving the meristematic identity of the leaf margin. The NAM-CUC transcription factor GOBLET determines boundaries within meristematic regions, that are necessary for organ initiation [30, 53], while the MYB transcription factor *CLAU* regulates the exit from the morphogenetic phase of tomato leaf development by affecting the CK / GA balance [26]. The changes in the expression levels of these genes upon treatment with bacilli isolated from high-CK genotypes (Fig. 6) further supports the notion that these particular bacilli isolates boost the leaf morphogenetic potential, in part through the promotion of CK signaling. Possibly, these effects are also mediated by bacterial CK produced by these bacilli isolates for the purpose of their interaction with the host plant they colonize, though further work is needed to examine the role of bacterial CKs in this interaction, and determine whether plant developmental programs can be, directly or indirectly, altered by bacterial CK.

Interestingly, age-related immunity was recently suggested to be microbiome dependent [14]. This raises the attractive possibility that the effect of CK on microbial content, which depends on plant age / developmental status, could also relate to CK-mediated immunity, i.e., CK-mediated immunity is age-dependent, or age-dependent immunity is CK-mediated, depending on the context. Further work is needed to elucidate the level of overlap between these two previously described phenomena.

Given its roles in growth and development, CK basically alters aging. In high CK content, plants become more morphogenetic, meristems are supported for longer times, senescence is delayed, and thus, "juvenility" is retained, i.e., "aging" is delayed. This delay apparently causes a lengthening of the developmental and temporal windows that support bacilli in the phyllosphere, leading to both increased growth and development (Figs. 3, 4, 5, 6, Additional file 1: S2-S3), and improved pathogen resistance [16, 22].

## Conclusion

Analyzing developmental-age related changes in the phyllosphere microbiome, we observed a developmental age associated decline in microbial richness and diversity, accompanied by a decline in the presence of growth promoting and resistance inducing bacilli in the phyllosphere. We show that this is likely caused by the parallel decline in CK content as the plant ages. Treating WT seedlings with bacilli isolated from high-CK genotypes, resulted in significant alterations to plant development, and increased agricultural productivity. This suggests that bacterial treatments, either as single isolate or in a consortia context, could be examined in order to "re-introduce" these beneficial microbial community members that are lost during aging, or prevent their loss from occurring. Additional work is needed to examine the performance of these bacilli in agricultural settings.

## Materials and methods

### Plant materials and sample collection

During the winter of 2018, tomato leaf samples were collected from a roofed net house, 2 mm nylon mesh net, in ARO, Volcani Institute, Rishon Lesion, Israel. For each sampling age, 10 plants were used. The middle left-hand lateral leaflet of leaves 5–6 from 2 plants was pooled to generate each sample, for a total of 5 biological replicates per developmental age. Genotypes used, all in the cv. M82 background, were as follows: M82 background line; *pBLS* >> *IPT7*, which contains elevated endogenous levels of CK- referred to hereinafter as "*pBLS* >> *IPT*" or "IPT"; *clausa*, which has increased CK sensitivity coupled with decreased CK content, referred to hereinafter as "*clausa*" or "*clau*"; and the CK depleted *pFIL* >> *CKX3*, referred to hereinafter as "*pFIL* >> *CKX*" or "CKX" [16].

### 16S rRNA amplification, amplicon sequencing and bioinformatic analysis

To examine whether plant developmental age affects tomato phyllosphere composition, phyllosphere microbial DNA was extracted from the phyllosphere of tomato (*S. lycopersicum* M82) seedlings (10 days post germination), vegetative plants (3 weeks post germination) and reproductive flowering plants (6 weeks post germination), grown in a net house in the winter of 2018. The study followed a randomized complete block design. Biological replicates from the 3 different developmental stages were transplanted into the net house at randomly interspersed locations, in three randomized blocks. All samples were collected when the oldest plants reached the "reproductive" stage at 6 weeks old, on the same sampling date. For phyllosphere DNA isolation,10 different plants were used. Five leaflet samples per sampling age were collected from the middle left-hand lateral leaflets of leaves 5–6, using ethanol-sterilized forceps. 5 biological replicates were prepared from the 10 different plants, and the 4 best samples in terms of DNA concentration and purity (as measured by nanodrop spectrophotometer) were used for amplicon preparation and sequencing, as described below. Twenty mL of 0.1 M potassium phosphate buffer at pH 8 were added to the tubes. The samples were sonicated in a water bath for 2 min and vortexed for 30 s twice. The pellet of microbes was obtained after centrifugation at 12,000*g* for 20 min at 4 °C. The pellet was re-suspended in potassium phosphate buffer [16]. Total DNA from tomato phyllosphere microorganisms was isolated using modified protocols described by Yang et al. [43] and Tian et al. [54], and used as a template for 16S rRNA PCR amplification. 16S rRNA amplicons were generated with the following primers:

CS1_515F:5′-ACACTGACGACATGGTTCTACAGTGCCAGCMGCCGCGGT-3′; CS2_806R: 5′-TACGGTAGCAGAGACTTGGTCTGGACTACHVGGGTWTCT-3′ [55]. Amplicon sequencing was conducted at the UIC core facility, using Illumina MiSeq paired-end sequencing. An average of 50,000 reads per sample were generated. QIIME 1.9 [56] was used for basic bioinformatics analysis. Read merging was conducted using PEAR [57]. Sequence trimming was done by trimming primer sequences and ambiguous end nucleotides from the reads. Reads shorter then 225 bp and reads with internal ambiguous nucleotides were discarded. ~ 70% of the reads passed trimming. Chimeric sequences were identified using the USEARCH algorithm [58] as compared with a reference database (silva_132_16S.97), and removed. Less than 5% of the reads on average were chimeric. Sub-OTU processing was done using a master cutoff of 10 and a cluster threshold of 0.97. Sequences were dereplicated and those with counts below the threshold were clustered. Clusters were annotated and a biological observation matrix (BIOM; sample-by-taxon abundance table) was generated. Taxonomy for the operational taxonomic units (OTUs) was assigned using BLAST against the Silva database [59] (silva_132_16S.97). About 60 phyla were identified. Alpha and beta-diversity, and Shannon index, were performed with QIIME 1.9 as well the workflow script core_diversity_analysis.py. The sequence data generated in this study was deposited to the Sequence Read Archive (SRA) at NCBI under PRJNA729221.

### Quantification of leaf bacteria through DNA qPCR

Total DNA extracts prepared as described above from the phylloshpere of plants grown in an ambient nethouse, were used for quantification of specific genes using qPCR. Total 16S rRNA and bacillus genes copy numbers were obtained using the 16S rRNA 515F/806R (primer efficiency 1.00) primer pair (515f: 5′-GTGCCAGCMGCCGCGGT-3′ and 806R: 5′-GGACTACHVGGGTWTCT-3′) [55] and bacillus specific BacF/BacF (primer efficiency 0.99) (BacF: 5′- AGGGTCATTGGAAACTGGG-3′ and 806R: 5′-CGTGTTGTAGCCCAGGTCATA-3′) [60], respectively. The quantification was performed with a Rotor-Gene Q machine (Qiagen) detection system and Power SYBR Green Master Mix protocol (Life Technologies, Thermo Fisher, United States). The PCR reaction was initiated at 95 °C for 3 min, followed by 40 three-step amplification cycles consisting of 10 s denaturation at 95 °C, 15 s annealing at 60 °C and 20 s extension at 72 °C. A final dissociation stage was run to generate a melting curve for verification of amplification product specificity. For 10 μl reaction mixture, 0.25 μM of forward and reverse primers were added to the PCR assay mixtures consisting of 5 μl Power SYBR Green Master Mix, 2.2 μl sterile nuclease-free water, and 2.0 μl template DNA. The standard regression curve was obtained using a *B. megaterium* 16S rRNA gene fragment and serial 1:10 dilutions (1 ng). Four replicates of each standard dilution were prepared to generate a mean value. The standard regression curve was prepared to determine the gene copy numbers in the unknown samples, and numbers were normalized to the standard sample. All PCR reactions were performed in triplicates.

### Bacterial isolate treatments

Epiphytic bacteria were isolated and identified as described [16]. Bacilli are well known to have growth-promoting effects. In our previous work, we characterized several bacilli isolates from different species obtained from the phyllosphere of *pBLS* >> *IPT*, a high CK content genotype, finding them to promote plant immunity and disease resistance [16]. The additional *Pseudomonas* and *Ralstonia* isolates used in this work are also phyllosphere isolates, and were used as Gram-negative control isolates, to examine whether observed phenomena are limited to particular taxons, or can be effected by bacteria from different phyla.

Accession numbers and details of bacterial isolates used in this study are provided in Table 1.

**Table 1:** Bacterial isolates used in this work

ID	Accession	Species	Source
4C	MZ148746	*Bacillus megaterium*	*pBLS* >> *IPT* isolate
R3C	MZ148747	*Ralstonia pickettii*	*pBLS* >> *IPT* isolate
R2E	MZ148745	*Bacillus pumilus*	*pBLS* >> *IPT* isolate
IN68	–	*Pseudomonas aeruginosa*	Wheat phyllopshere isolate obtained from Jonathan Friedman, Hebrew University of Jerusalem
SB491	SB491 "legacy" strain [61, 62]	*Bacillus subtilis*	Jonathan Friedman, Hebrew University of Jerusalem

*S. lycopersicum* cv. M82 seeds were sown after surface sterilization (with 1.5% NaOCl for five minutes, followed by three rinses with sterile water) in a tray containing germination mixture consisting of 40% peat, 40% coconut husks and 20% quartz (Green90, Even Ari Green, Israel). After germination, a single tomato seedling was transplanted to each pot (0.5 L, diameter = 10 cm) containing potting mixture consisting of 70% peat and 30% porous tuff 0–8 mm in size (Green332, Even-Ari Green, Israel). Pots were kept in the nethouse at ambient temperature (Day—20–26°C; Night—12–20°C), 12-h photoperiod. Bacterial colonies of *B. pumilus* R2E, *B. megaterium* 4C, *R. pickettii* R3C, *P. aeruginosa* IN68 and *B. subtilis* SB491 from a 24 h plate (Luria–Bertani medium) culture were washed twice in sterile distilled water, and then re-suspended in a 10 mM MgCl_2_ solution. The cell suspension was adjusted to an optical density of OD_600_ = 0.1 (approximately equal to 10^8^ CFU mL^−1^) using a spectrophotometer (Tecan Spark multimode plate reader). For mature plants (4–5 weeks old at the start of the experiment), soil drenching of the plants was carried out by pouring 10 mL of bacterial suspension into each pot once a week, for four weeks. Growth parameters were measured as previously described by Pizarro et al. [63]. Plant weight was determined by weighing only the vegetative tissue (after harvesting the fruits) without the roots. Total soluble sugars were measured as BRIX percentage on a digital refractometer with a range of BRIX 0–85% ± 0.2%, from a random sample of 3–4 fruits per plant. Harvest index (HI) was calculated as the ratio between the total yield and total biomass. For seedlings, plants were spray-drenched using a hand-held spray bottle once a week, for two weeks, starting from cotyledon emergence. Plants treated with sterile distilled water served as controls.

### Plant RNA preparation and qRT-PCR

RNA was isolated from liquid N_2_ ground shoot apices of 10 day old seedlings, including the shoot apical meristem (SAM) and P1-P5 leaf primordia, of 12 to 15 seedlings individually treated with bacteria viz., *B. pumilus* R2E, *B. megaterium* 4C, *R. pickettii* R3C, and *P. aeruginosa* IN68, using TRI reagent (Sigma Aldrich) as per the manufacturer’s recommendations. RNA concentrations were quantified, and cDNA was then synthesized from 2 μg RNA in a 20 μL reaction, using both reverse transcriptase and oligo(dT) primers provided with the cDNA Synthesis kit (Promega, United States). RT-qPCR was performed according to the Power SYBR Green Master Mix protocol (Life Technologies, Thermo Fisher, United States), using a Rotor-Gene Q machine (Qiagen) detection system. The PCR reaction was initiated at 95 °C for 3 min, followed by 40 three-step amplification cycles consisting of 10 s denaturation at 95 °C, 15 s annealing at 60 °C and 20 s extension at 72 °C. A final dissociation stage was run to generate a melting curve for verification of amplification product specificity. For 10 μl reaction mixture, 0.25 μM of forward and reverse primers were added to the PCR assay mixtures consisting of 5 μl Power SYBR Green Master Mix, 2.2 μl sterile nuclease-free water, and 2.0 μl template DNA. Primer sequences used for the qRT-PCR analyses are detailed in Additional file 1: Table S1 [22, 30]. For developmental gene analysis, we chose genes related to boundary definition, which is important for organ initiation [30, 52], meristem maintenance, which is important for increased morphogenesis [26], and, given the *TCSv2* activation results, genes of the CK pathway. Expression of all assayed genes was normalized relative to a geometric mean of the copy number of the three tomato housekeeping genes, ribosomal protein *SlRPL8* (Solyc10g006580), *Slcyclophilin* (Solyc01g111170) and *SlEXP* (Solyc07g025390). All primer efficiencies were in the range 0.98–1.03 (see Additional file 1: Table 1). Relative expression was calculated using the copy number method for gene expression [64].

### Seedling developmental analysis, dissection, and imaging

Seedlings were harvested from soil by cutting them at the stem base. Height from the stem base to the SAM, and fresh weight were measured using a ruler and an analytical scale, respectively. For developmental analyses, we chose to examine leaf development, which follows a predictable and well characterized program *in S. lycopersicum* M82 [26, 29], in depth. For this, we selected the *B. megaterium* isolate 4C, which consistently performed best in growth promotion assays we conducted (Fig. 4). We conducted an in depth analysis of leaf complexity, starting from the third leaf primordium (p3), in mock plants and plants treated with 4C. P3 was chosen since the first and second primordia are completely un-patterned in M82 [30]. The number of leaves was counted by dissecting the shoot under a stereomicroscope and counting all the initiated leaves, starting from P1. Differentiation of the meristem to floral and sympodial follows a predictable pattern in *S. lycopersicum* M82 [52, 65], and was analyzed microscopically in dissected shoots. Leaves are produced successively on the plant, and at a given time point each leaf is at a different developmental stage. Each leaf is thus characterized by both its position on the plant (for example, L1 is the first leaf produced and L5 is the fifth), and by its developmental stage. Thus, L5 P1 is the fifth leaf when it is at the P1 stage and has just initiated from the SAM, and it becomes L5 P2 after the next primordium initiates, and so on. For each developmental stage analyzed, the fifth leaf from at least ten different plants was analyzed for leaf complexity (the amount of leaflets).

CK signaling is mediated via a two-component multistep phosphorelay cascade. The TCS sensor was designed using the conserved DNA binding domain in the promoter of type A response regulators (RRs) that is recognized by type-B response regulators. The CK sensor TCSv2 was created based on the CK phosphorelay network, using elements derived from the conserved DNA binding domain in type A RRs [52]. For TCSv2:3XVENUS, the DNA sequence of TCSv2 was synthesized with flanking NsiI and BamHI restriction site [30] and the synthetic promoter was then cloned adjacent to 3xVENUS-N7 in the pBJ36 vector [52]. The construct was subcloned into the pGREEN binary vector. For analysis of TCSv2:3XVENUS expression, dissected whole-leaf primordia were placed into drops of water on glass microscope slides and covered with cover slips. The pattern of VENUS expression was observed with a Nikon SMZ-25 stereomicroscope equipped with a Nikon-D2 camera and NIS Elements v. 5.11 software [52].

### Data analysis

All experimental data is presented as minimum to maximum values with median or mean, in boxplots or floating bars, or as average ± SEM, with all points displayed. For microbiome analyses, differences between two groups were analyzed for statistical significance using a Mann–Whitney test, or a two-tailed *t*-test, with Welch's correction where applicable (unequal variances). Differences among three groups or more were analyzed for statistical significance with a Kruskal–Wallis Test, with Dunn's multiple comparisons post-hoc test. For all other analyses, differences between two groups were analyzed for statistical significance using a two-tailed *t*-test, with Welch's correction where applicable (unequal variances), and differences among three groups or more were analyzed for statistical significance with a one-way ANOVA. Regular ANOVA was used for groups with equal variances, and Welch’s ANOVA for groups with unequal variances. When a significant result for a group in an ANOVA was returned, significance in differences between the means of different samples in the group were assessed using a post-hoc test. The Tukey test was employed for samples with equal variances when the mean of each sample was compared to the mean of every other sample. The Bonferroni test was employed for samples with equal variances when the mean of each sample was compared to the mean of a control sample. The Dunnett test was employed for samples with unequal variances. All statistical analyses were conducted using Prism^8^ software (Graphpad, San Diego, Calif).

## Supplementary Information

**Additional file 1: Figure S1:** The amount of bacteria in the phyllosphere is CK dependent. **Figure S2:** *B. megaterium* 4C induces differentiation of the flowering meristem. **Figure S3:** *B. megaterium* 4C and *B. pumilus* R2E accelerate leaf development‐ Leaf complexity over time. **Table S1:** qRT-PCR primers used in this work.

## Data Availability

The authors declare that the data supporting the findings of this study are available within the paper and its Supplementary information files. Raw data is available through NCBI-SRA, Bioproject PRJNA729221.
