# Supplementary material for: Cytokinin-microbiome interactions regulate developmental functions
Source: Environ Microbiome. 2022 Jan 15;17:2. doi: 10.1186/s40793-022-00397-2 (PMC8760676; doi:10.1186/s40793-022-00397-2)
Supplement: Supplementary file 1 — Additional file 1: Figure S1: The amount of bacteria in the phyllosphere is CK dependent. Figure S2: B. megaterium 4C induces differentiation of the flowering meristem. Figure S3: B. megaterium 4C and B. pumilus R2E accelerate leaf development‐ Leaf complexity over time. Table S1: qRT-PCR primers used in this work. [file 40793_2022_397_MOESM1_ESM.pdf]

Supplemental materials

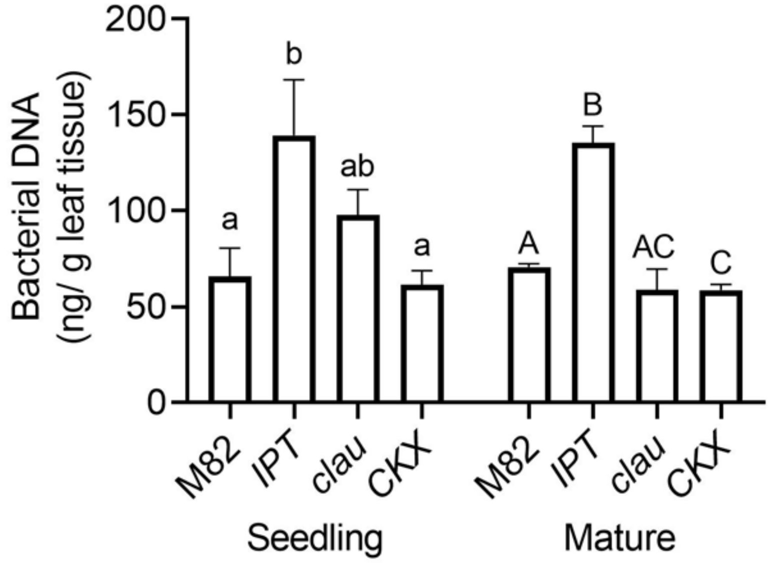

Figure S1. The amount of bacteria in the phyllosphere is CK dependent.
Bacterial DNA was extracted from indicated genotypes at the seedling and mature plant stages. Amount of bacterial DNA obtained per gram leaf tissues is plotted. Graphs depict mean ±SE. Different letters indicate statistically significant differences in an unpaired two-tailed t-test with Welch's correction, N=10, $p<0.05$.

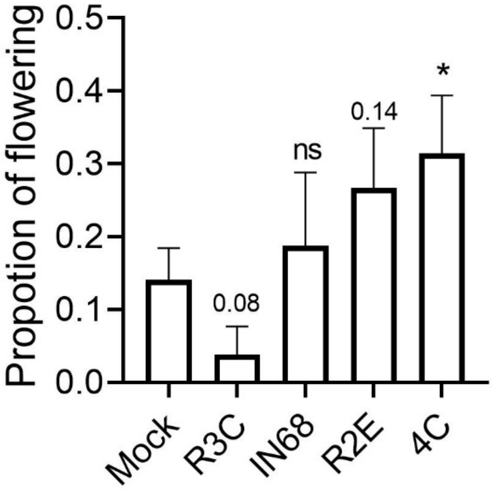

Figure S2: ***B. megaterium*** **4C induces differentiation of the flowering meristem.**
Presence of the floral meristem was examined in 10 day old M82 mock and bacterial isolate treated seedlings. Graphs depict mean ±SE. Five independent experiments were conducted, N=30. Asterisks represent statistical significance from mock treatment in a two-tailed t-test. *p<0.05.

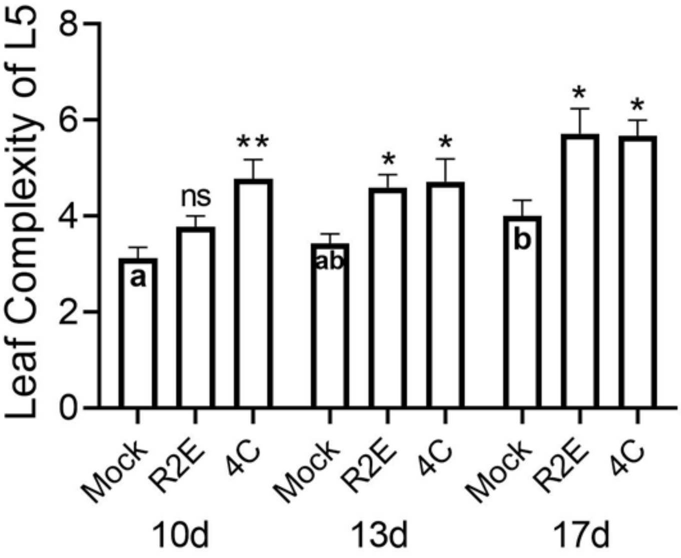

Figure S3: *B. megaterium* 4C and *B. pumilus* R2E accelerate leaf development- Leaf complexity over time.

Leaf complexity of the fifth leaf (L5) was measured in M82 mock, R2E, and 4C treated plants over time. Bars depict mean ±SEM. Three independent experiments were conducted, N=10 for each time point. Asterisks represent statistical significance from mock treatment, and different letters represent statistically significant differences among samples, in a two-tailed t-test. *p<0.05, **p<0.01.

Supplemental Table1 Primers used for qRT-PCR.

**Gene**	**Accession No.**	**Primer pairs (5'-3')**	**Efficiency**
PR1a (Pathogenesis related-1a)	Solyc01g106620	F: CTGGTGCTGTGAAGATGTGG<br>R: TGACCCTAGCACAACCAAGA	0.98
LoxD	Solyc03g122340	F: CCATCCTCACCACCCTCATC<br>R: TACTCGGGATCGTTCTCGTC	0.97
IPT3	Solyc01g080150	F: TTCCATGCTTGATGTGCTTC<br>R: GCTTGCTGTCAACGTCAAAA	0.98
CKX2	Solyc01g088160	F: CCCCGAAAATGGTGAAATG<br>R: CAAAGTGGCTTGCTTGAACA	1.01
TKN2	Solyc02g081120	F: CCATATCCATCGGAATCTCAG<br>R: TGGTTTCCAATGCCTCTTTC	1.00
CKX5	Solyc04g016430	F: TGTCACTGGTAAAGGAGAGGTG<br>R: GAGCAATCCTAGCCCTTGTG	1.02
CKX6	Solyc12g008900	F: CAGGTGCTAAGCCATACTCTAGG<br>R: GGACATTCCATTAGGGGACA	1.03
CLAU	Solyc04g008480	F: CCTCTCACAACAAGCAATGAACTT<br>R: AGGACGATGCAATGAGAGAGAC	0.97
GOBLET	Solyc07g062840	F: CAGGAGTTCGAAGGACGAGTGG<br>R: TTGGCTGTAGTGTATGCAAGGTG	1.00
TRR3/4	Solyc05g006420	F: CGTCCCCTAAAGCATTCTCA<br>R: CGTCTTGTTGGTGATGTTGG	0.98
EXP (Expressed)	Solyc07g025390	F: TGGGTGTGCCTTTCTGAATG<br>R: GCTAAGAACGCTGGACCTAATG	1.00
RPL8 (Ribosomal protein L2)	Solyc10g006580	F: TGGAGGGCGTACTGAGAAAC<br>R: TCATAGCAACACCACGAACC	1.03
CYP (Cyclophilin)	Solyc01g111170	F: TGAGTGGCTCAACGGAAAGC<br>R: CCAACAGCCTCTGCCTTCTTA	1.03
